# Supplementary material for: Does coronavirus disease 2019 history alone increase the risk of postoperative pulmonary complications after surgery? Prospective observational study using serology assessment
Source: PLoS One. 2024 May 21;19(5):e0300782. doi: 10.1371/journal.pone.0300782 (PMC11108156; doi:10.1371/journal.pone.0300782)
Supplement: S1 Table — (DOCX) [file pone.0300782.s001.docx]

| **S1 Table. Baseline characteristics and outcomes.** | | | |
| --- | --- | --- | --- |
|  | **History of COVID-19**  **(N = 100)** | **No history of COVID-19** | |
|  |  | **Seronegative**  **(N = 100)** | **Seropositive**  **(N = 44)** |
| Age | 54.84 (±15.04) | 58.20 (±14.06) | 57.82 (±15.13) |
| Male | 46 (46.0) | 43 (43.0) | 25 (56.8) |
| Body mass index | 24.90 (±3.84) | 24.50 (±3.96) | 24.41 (±3.17) |
| ASA physical status |  |  |  |
| I | 6 (6.0) | 1 (1.0) | 4 (9.1) |
| II | 79 (79.0) | 84 (84.0) | 31 (70.5) |
| III | 15 (15.0) | 15 (15.0) | 9 (20.5) |
| Non-smoker | 83 (83.0) | 80 (8.0) | 31 (70.5) |
| Ex-smoker | 8 (8.0) | 10 (1.0) | 2 (4.5) |
| Current smoker | 9 (9.0) | 10 (10.0) | 11 (25.0) |
| Comorbidities |  |  |  |
| Hypertension | 32 (32.0) | 35 (35.0) | 15 (34.1) |
| Diabetes mellitus | 13 (13.0) | 21 (21.0) | 8 (18.2) |
| Stroke | 8 (8.0) | 4 (4.0) | 2 (4.5) |
| Coronary artery disease | 4 (4.0) | 3 (3.0) | 2 (4.5) |
| Chronic kidney disease | 1 (1.0) | 8 (8.0) | 2 (4.5) |
| Heart failure | 0 | 1 (1.0) | 0 |
| Chronic obstructive pulmonary disease | 1 (1.0) | 2 (2.0) | 2 (4.5) |
| Tuberculosis | 1 (1.0) | 1 (1.0) | 2 (4.5) |
| Malignancy | 18 (18.0) | 23 (23.0) | 7 (15.9) |
| Operation duration | 226.05 (±142.83) | 195.58 (±123.73) | 174.20 (±87.29) |
| Operation types |  |  |  |
| Orthopedic | 10 (10.0) | 5 (5.0) | 3 (6.8) |
| Head and neck | 7 (7.0) | 5 (5.0) | 0 |
| Abdominopelvic | 21 (21.0) | 15 (15.0) | 7 (15.9) |
| Vascular | 1 (1.0) | 3 (3.0) | 1 (2.3) |
| Brain | 61 (61.0) | 72 (72.0) | 33 (75.0) |
| Postoperative pulmonary complication | 24 (24.0) | 26 (26.0) | 12 (27.3) |
| Atelectasis | 8 (8.0) | 7 (7.0) | 5 (11.4) |
| Pleural effusion | 7 (7.0) | 10 (10.0) | 2 (4.5) |
| Pulmonary edema | 1 (1.0) | 2 (2.0) | 0 |
| Pneumonia | 4 (4.0) | 4 (4.0) | 1 (2.3) |
| Aspiration pneumonitis | 0 | 1 (1.0) | 0 |
| Need for oxygen therapy | 18 (18.0) | 15 (15.0) | 10 (22.7) |

Data are presented as mean (±standard deviation) or number (%). ASA, American Society of Anesthesiologists; COVID, Coronavirus Disease.
